# Commission for Investigating Yellow Fever Inoculation

**Published:** 1886-02-20

**Authors:** Domingos Freire

**Affiliations:** No. 29 Cattete street; Rio de Janerio


### COMMISSION FOR INVESTIGATING YELLOW FEVER INOCUATION.

The following translation of a letter from Dr. Domingos Freire to Dr. J. McF. Gaston of this city, has, at our request, been furnished us by the latter, and is given to the medical public with a firm conviction that our readers may rely upon the important statement of the writer, showing the exemption of 6,000 inhabitants of Rio de Janeiro from yellow fever, who had been inoculated, while five hundred deaths occurred among those who were not inoculated, up to the close of the past year. It matters not practically what may be the precise mode of investigation that has been adopted, nor the exact place that this germ may occupy in the bacterial family, if a prophylactic process has been discovered by which the human race are secured against the development of yellow fever, when exposed to agencies which would otherwise produce the disease in the individual. That such a result has been obtained in Brazil, Mexico, and Panama by the same means, affords a strong corroborative evidence of the trustworthiness of the process adopted in these several localities, and certainly raises a strong presumption in favor of verifying the correctness of the inferences, which seem to indicate the applicability of this protective inoculation in our Southern seaports.

Should there remain a doubt in regard to the facts, a close scrutiny of the data presented in the field of these experiments should lead to satisfactory conclusions by the proposed Commission, and it cannot fail to serve as a crucial test of the virtues of this process of inoculation with attenuated cultures of the microbia virus of yellow fever. The sanction of the Brazilian Government for this undertaking originally, and the proceedings instituted and prosecuted during these past six years, under the authoritative supervision of those in power, with the final details published in the French and English languages, as well as in the Portuguese idiom of the country—giving the successful results of the methods adopted by Dr. Domingos Freire—indicate all the conditions of authentic and genuine scientific developments.

The facts concerning this investigation, which were known to Dr. Gaston while still residing in Brazil, and trustworthy reports of its progress since he left that Empire, in 1883, from sources altogether independent of Dr. Freire, led him to the conclusion that a veritable discovery had been made, which ought to be applied in this country for the prevention of yellow fever. He accordingly went to work through the regular official channels of the United States Government, and ultimately by addressing a communication to Dr. Joseph Holt, president of the Louisiana State Board of Health, has been encouraged to hope for a realization of his proposition for an investigation by a commission, authorized by Congress, to be appointed by the President of the United States. This work of such momentous practical bearings upon the welfare of our people, and in the interest of true scientific research, would, if entrusted to him, be faithfully executed ; and we trust Dr. Gaston may be assigned a place on the commission :

RIO DE JANERIO, December 27, 1885.

DEAR DOCTOR GASTON : It is with great satisfaction that I reply to your kind letter just received, to which I hasten my answer.

The lively interest that you have shown in presenting my discovery to the public is so gratifying to me that I cannot find words which express adequately my deep sense of obligation.

I have entire confidence that, under the protection of your important name, my discovery of the prevention of yellow fever by means of inoculation with attenuated cultures, will be shortly extended throughout the grand American Republic heretofore devastated by that scourge. The day on which this happens will be that of the richest reward for the great labor and hard sacrifices which I have undertaken in the fulfillment of this humane problem ; and your name will be found linked with this work of Christian charity and progressive civilization.

I have read with all attention your correspondence, as likewise the authoritative opinion of Dr. Holt, to whom I write at present date. His ideas will most assuredly be duly weighed by the American Association of Public Health and I judge that the commission of medical men of which you spoke will come very soon to give us the distinguished honor of verifying the results of our prophylactic measure, which, up to the present time, has been attended with such notable success that not one of the six thousand who were inoculated during the current year have died of yellow fever, while nearly five hundred who were not inoculated have sunk into the grave as victims of that disease.

In a short time I will send you a pamphlet, in which I give minute information upon the inoculations practiced, by which all may properly estimate the confidence which ought to be deposited in the preventive method that I propose.

I am, dear sir, with profound respect, your obedient servant,

DOMINGOS FREIRE,
No. 29 Cattete street.

